# Modelling of a Hall Effect-Based Current Sensor with an Open Core Magnetic Concentrator

**DOI:** 10.3390/s18041260

**Published:** 2018-04-19

**Authors:** Ivan Yatchev, Mehmet Sen, Iosko Balabozov, Ivan Kostov

**Affiliations:** 1 Faculty of Electrical Engineering, Tehcnical University of Sofia, 1000 Sofia, Bulgaria; mehmetsen@uludag.edu.tr (M.S.); i.balabozov@tu-sofia.bg (I.B.); igkostov@tu-sofia.bg (I.K.); 2 Vocational School of Technical Sciences, Uludağ University, 16059 Nilufer/Bursa, Turkey

**Keywords:** current sensors, Hall effect, 3D FEM, magnetic concentrator

## Abstract

The present paper deals with the modelling of a Hall effect current sensor with open core magnetic concentrator. 3D magnetic field modelling is carried out using the finite element method (FEM) and Comsol Multiphysics software. Two rectangular core constructions are considered. Different geometric parameters of the magnetic concentrator are varied and their influence on the sensor characteristic is studied, with the aim of reducing the dependence on the output signal on the distance to the conductor. Of the studied parameters, core window length leads to the most significant change in the sensor characteristic. Future work can include the optimization of the sensor construction.

## 1. Introduction

Current sensors based on the Hall effect are widely used in different applications and are the subject of increasing interest in recent years [1,2,3,4]. The output signal of sensors without a magnetic core strongly depends on the distance between the conductor and the Hall sensor, and different approaches are used for eliminating this dependence [5,6,7]. Where possible, constructions with magnetic cores are used [8,9]. Commercially available current measuring apparatus based on Hall effect sensors usually include a closed magnetic core with a small gap where the Hall sensor is placed. In some specific cases, though, the employment of closed cores as magnetic concentrators is not possible due to the construction of the system where the current to be measured flows.

Different geometries of Hall sensors working in current mode are studied in [10]. To prove the accuracy of COMSOL models, they make comparison with results achieved from a model implemented in Verilog-A software. In [11], various schemes of magnetic biosensors that are used for the identification of a wide spectrum of biological, physical, and chemical agents are presented. By numerical simulations using the finite element method (FEM), bending-induced stress effects on ultrathin cross-shaped magnetic sensors are analyzed in [12]. One application of this type of sensors is electronic or tactile skin. In [13,14,15], different arrays of Hall sensors working in current mode are studied, and electronic interfaces that are used with sensors are presented. To improve the characteristics of the magnetic sensors in [16,17], optimal design solutions are studied and an optimal geometry of the Hall plate is proposed.

In fixed arrangements, the sensor could be easily calibrated for any type of magnetic concentrator and also for coreless design. In nonfixed arrangements, though, especially in the case when a closed core concentrator is not practically realizable, open core magnetic concentrators could be employed, thus reducing the error in the current measurement due to an error in the distance measurement.

In the present paper, 3D FEM modelling of the magnetic field in a Hall effect-based current sensor with an introduced magnetic concentrator of open core type is carried out with the aim of reducing the dependence of the output signal on the distance between the conductor and the Hall plate. This can give the opportunity of further design of a current sensor with preliminary estimated error due to the deviation of the conductor position.

## 2. Studied Constructions

Two constructions of the magnetic core of the current sensor are considered: a rectangular C-core with one air gap for the Hall sensor (referred to as construction with one air gap or concentrator with one air gap), and a rectangular magnetic core with two air gaps: one for the Hall sensor and one additional air gap, placed on the opposite leg (referred to as construction with two air gaps or concentrator with two air gaps).

### 2.1. Construction with One Air Gap

**Figure 1:**
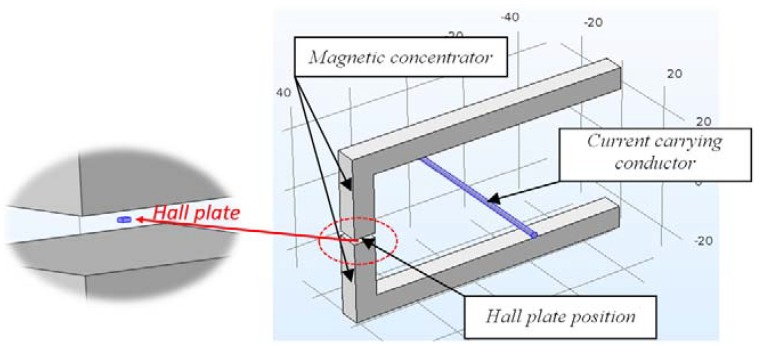
Construction with one air gap.

The construction consists of two ferromagnetic legs, which on the one end are linked with a third leg with an air gap, where the Hall plate is placed (Figure 1). The dimensions given in the figure are in millimeters. The Hall plate is also included in the model (inside the air gap) and its dimensions are considered to be 0.24 × 0.24 × 0.1 mm.

### 2.2. Construction with Two Air Gaps

**Figure 2:**
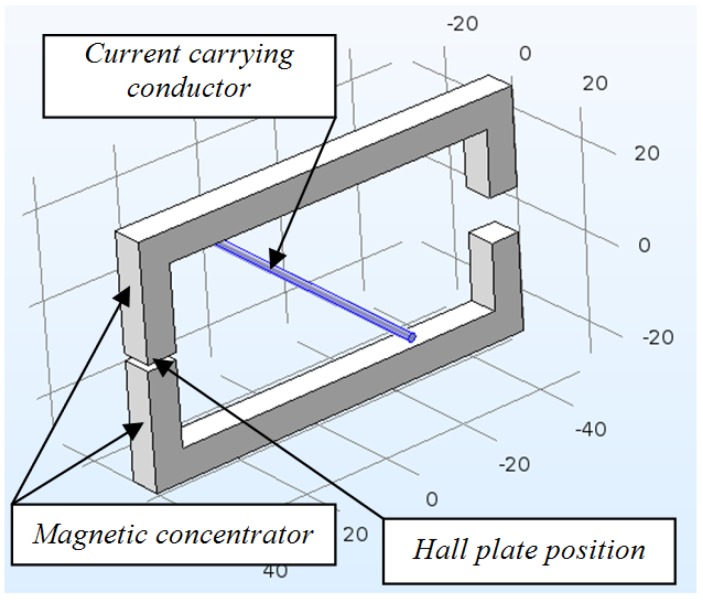
Construction with two air gaps.

The construction with two air gaps (Figure 2) is similar to the one in Figure 1, but a fourth leg with a second gap is introduced. The second gap is greater than the one where the Hall plate is located and is large enough in order to allow passing a conductor through it.

## 3. FEM Modelling

The finite element method is used for modelling the magnetic field of the two constructions. The Comsol Multiphysics program [18] has been employed for this purpose. The AC/DC module and Magnetic Fields and Electric Currents interfaces have been used. For computation of the Hall plate output voltage, the approach employed in [19] has been used.

**Figure 3:**
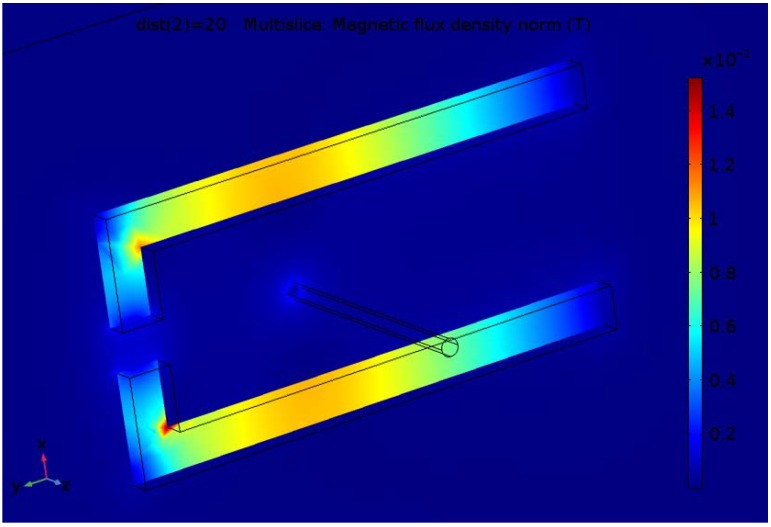
Flux density distribution for the construction with one air gap.

**Figure 4:**
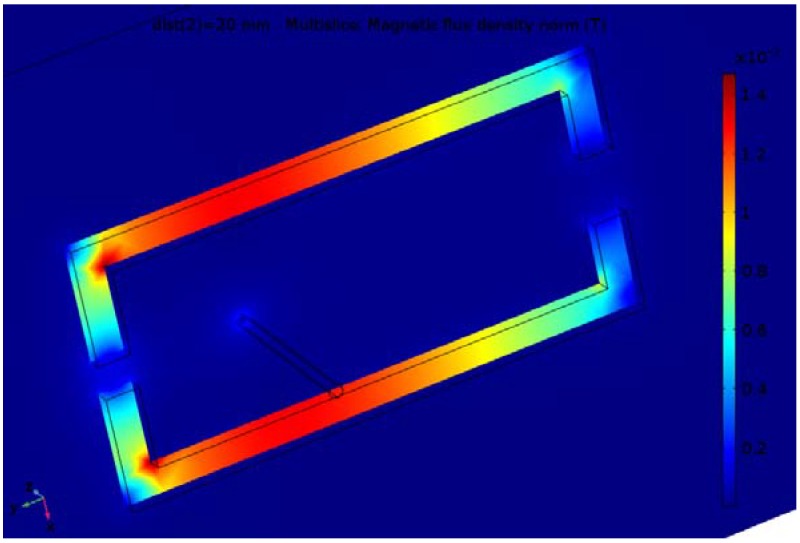
Flux density distribution for the construction with two air gaps.

The finite element analysis is carried out for the 10 A value of the current in the conductor and different parameters of the magnetic concentrator. Results for the magnetic flux density distribution for one variant of the two considered constructions are shown in Figure 3 and Figure 4.

Although the inner dimensions of the concentrator with two gaps is greater than for the one with one gap, the flux density is greater due to introducing the leg with a second gap.

**Figure 5:**
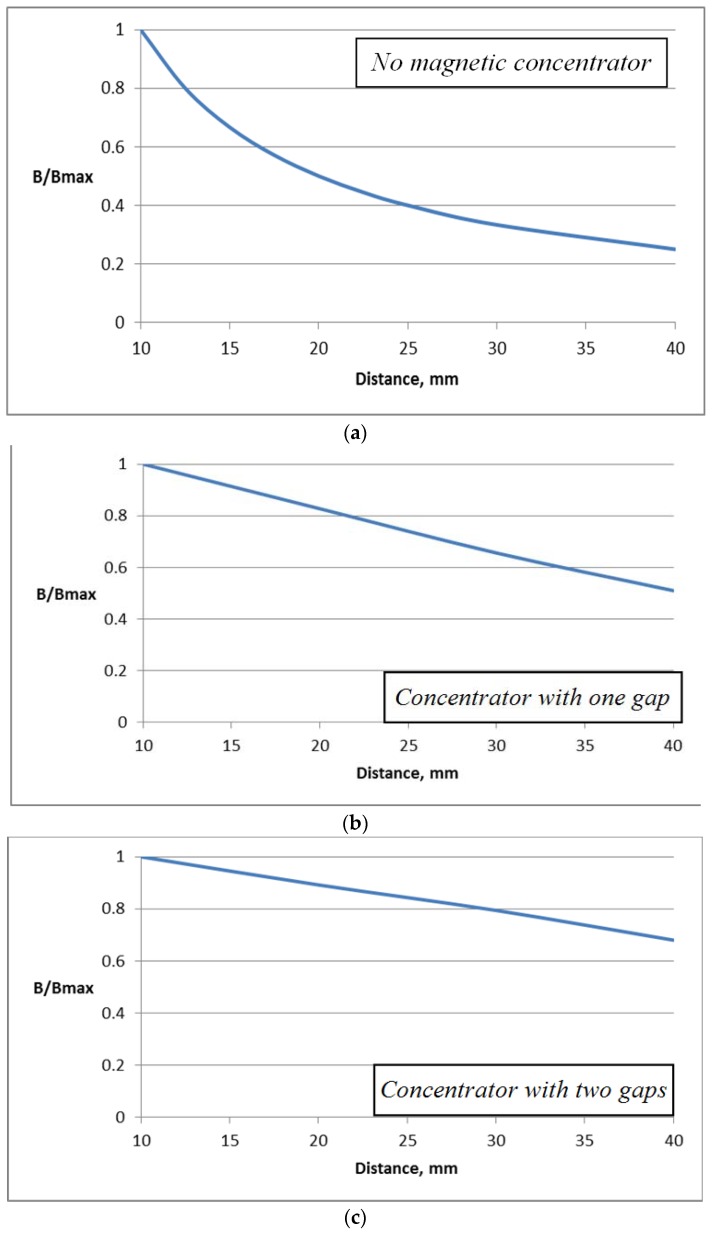
Dependence of the flux density on the distance to the conductor (**a**) no magnetic concentrator; (**b**) concentrator with one gap; (**c**) concentrator with two gaps.

In order to estimate the effect of introducing the magnetic concentrator, the dependence of the magnetic flux density in the Hall plate (volume integral average) on the distance to the conductor for construction without a magnetic concentrator is shown in Figure 5a. In Figure 5b,c, the same dependence is shown for the constructions with concentrators with one and two gaps. Both relationships are close to a linear one, while for the coreless design (without concentrator) the dependence is strongly nonlinear.

Relative values of the flux density are used in order to allow comparison between the different curves.

## 4. Results for the Influence of Different Parameters

**Figure 6:**
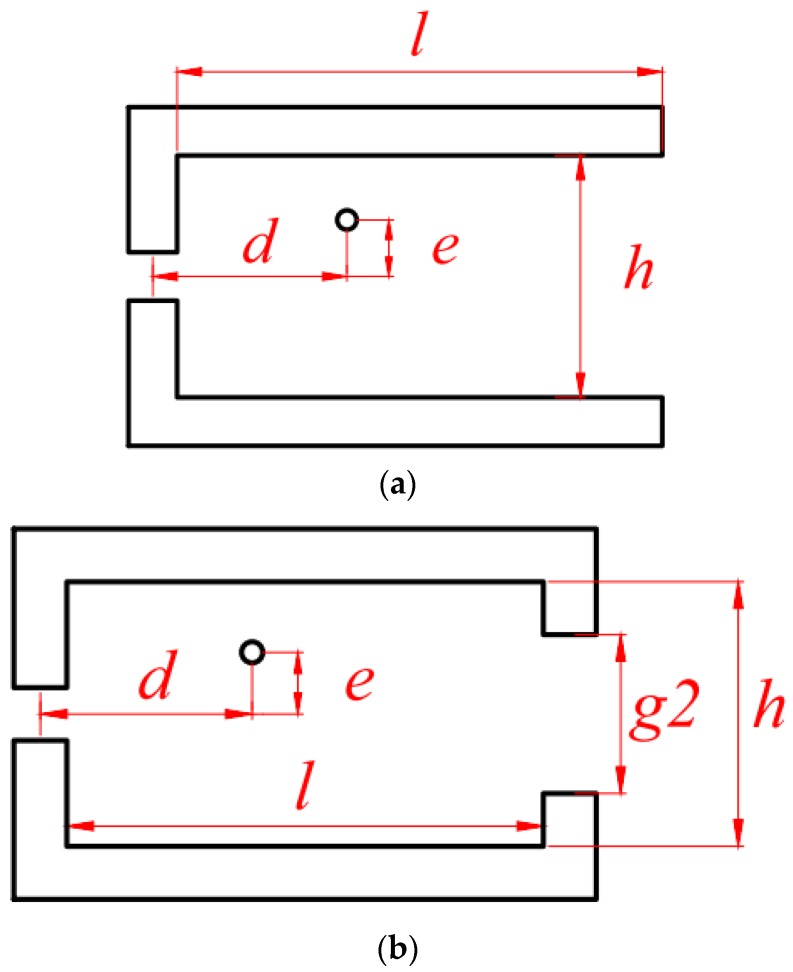
Parameters subject to variation for the two constructions: (**a**) construction with one air gap; (**b**) construction with two air gaps.

The influence of different geometric parameters has been studied for both constructions. The varied parameters are shown in Figure 6a,b. In addition to the distance between the Hall plate and the conductor (denoted by d), the parameters subject to variation are window length (l), window height (h), and eccentricity (e) of the conductor with respect to the central line. For the case of the concentrator with two gaps, the size *gap2* of the additional air gap is also varied.

The other geometric parameters are fixed, as follows:

- core cross section is of size 7 × 7 mm;
- air gap accommodating the Hall plate is 1.5 mm;
- conductor diameter is 2 mm.

### 4.1. Construction with One Air Gap

Three parameters have been varied for the construction with one air gap: the length *l* and height *h* of the window and deflection of the conductor from the central symmetry line *e*.

**Figure 7:**
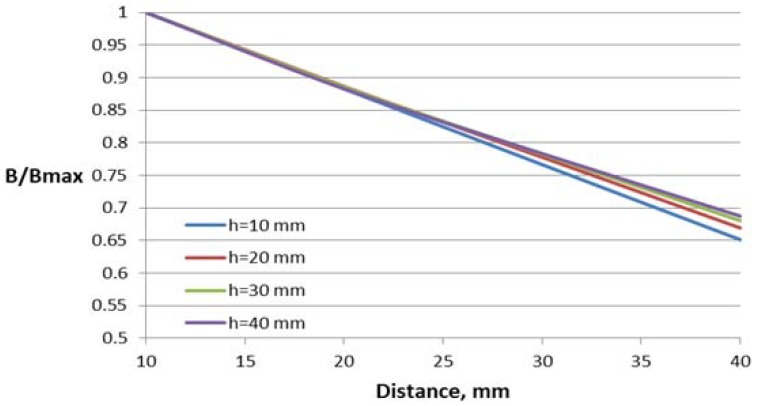
Dependence of the flux density on the distance to the conductor for the concentrator with one gap and different window lengths *l*.

**Figure 8:**
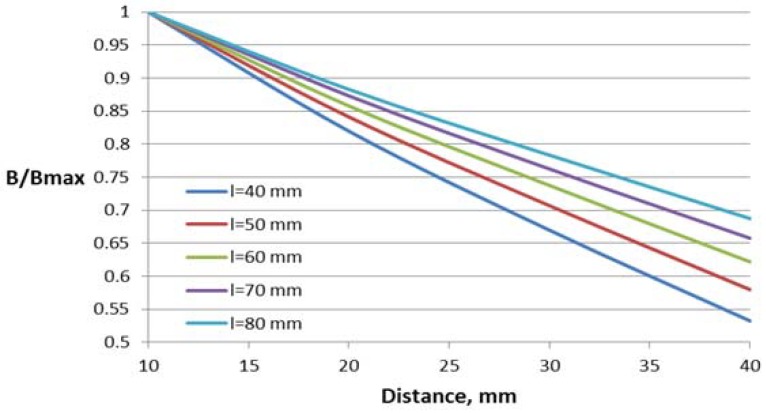
Dependence of the flux density on the distance to the conductor for the concentrator with one gap and different window heights *h*.

**Figure 9:**
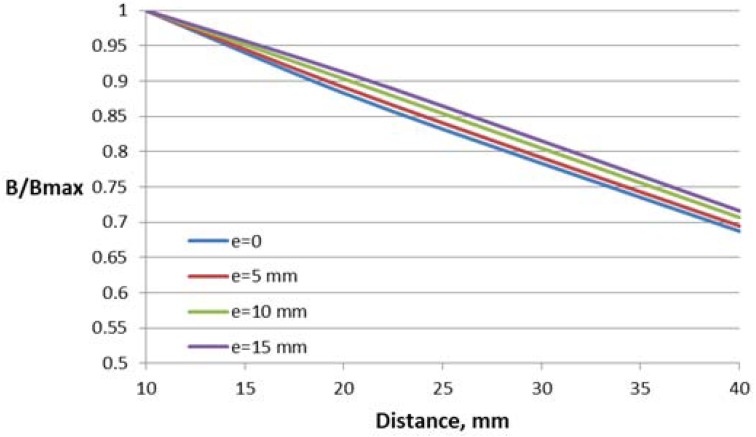
Dependence of the flux density on the distance to the conductor for the concentrator with one gap and different conductor deflections in the transversal direction *e*.

The dependence of the magnetic flux density B in the Hall plate (volume integral average) on the distance between the conductor and the Hall plate for different parameter values are shown in Figure 7, Figure 8 and Figure 9. For better comparison of the different characteristics, the flux density in the Hall plate is presented in relative units with respect to its maximal value Bmax, which is obtained in the plate at the minimal considered distance (10 mm) between the conductor and the Hall plate.

Core window length has a significant influence on the flux density–distance relationship, leading to about 30% difference between the flux density for the first and the last value of the length at the largest distance.

Variations of the core window height and deflection of the conductor from the central symmetry line do not lead to significant change in the characteristics (about 4–6% at the largest distance).

### 4.2. Construction with Two Air Gaps

Four parameters have been varied for the construction with two air gaps. In addition to the parameters varied for the case of one air gap, the value of the additional air gap is also varied. 

**Figure 10:**
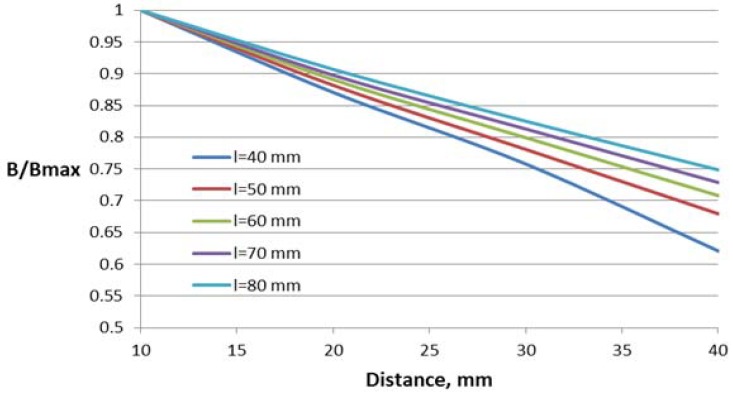
Dependence of the flux density on the distance to the conductor for the concentrator with two gaps and different window lengths *l*.

**Figure 11:**
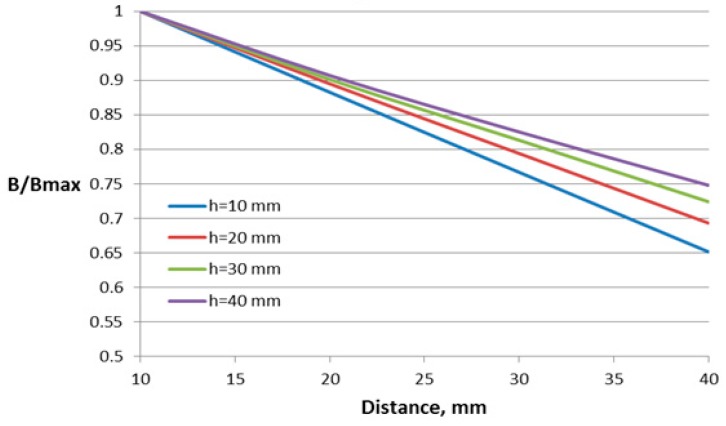
Dependence of the flux density on the distance to the conductor for the concentrator with two gaps and different window heights *h*.

**Figure 12:**
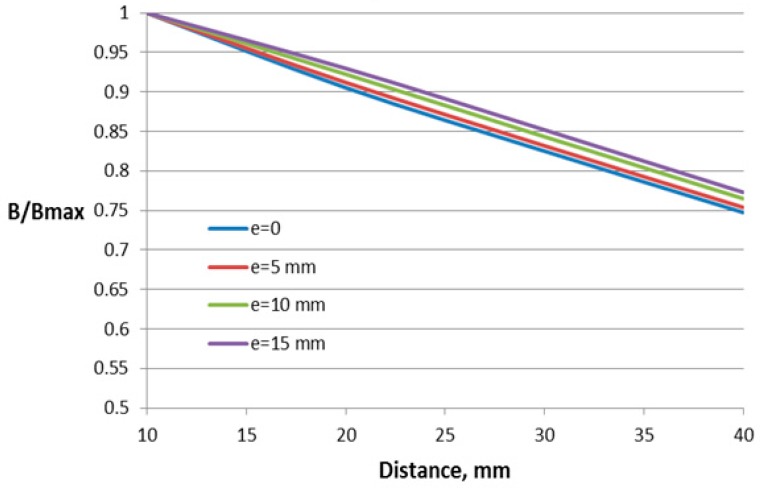
Dependence of the flux density on the distance to the conductor for the concentrator with two gaps and different conductor deflections in the transversal direction *e*.

**Figure 13:**
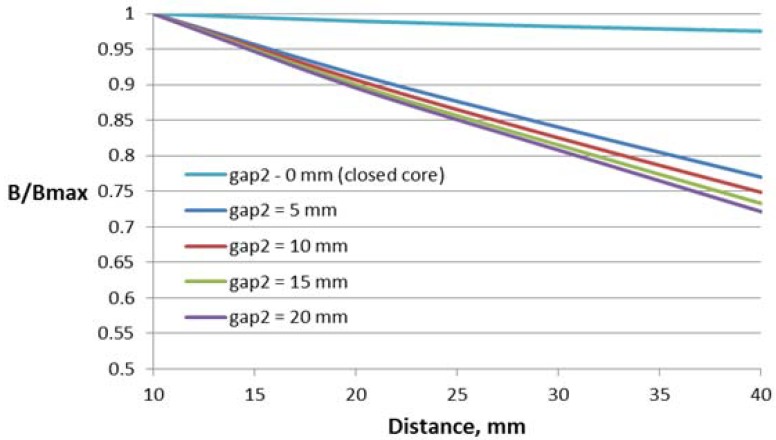
Dependence of the flux density on the distance to the conductor for the concentrator with two gaps and different values of the second air gap (gap2).

The dependences of the relative flux density on the distance between the conductor and Hall plate for different parameters of this construction are shown in Figure 10, Figure 11, Figure 12 and Figure 13.

For this construction, both sizes of the core window (length and height) influence the sensor performance. Regarding the influence of the second air gap, as it can be expected, the lowest dependence on the distance features the construction with the smallest value of the second air gap. For comparison, in Figure 13, the dependence of the construction with the closed core is also given.

For this construction, the window length and height have more influence on the dependence on the conductor position, leading to changes of 13% and 10%, respectively. The variation of the other two studied parameters results in less than 5% change in the characteristic.

All the obtained characteristics with open core magnetic concentrators, though, feature an almost linear dependence on the distance between the conductor and the Hall plate. 

### 4.3. Results for Different Values of the Measured Current

For estimation of the influence of the value of the current on the output voltage signal from the Hall plate, the output signal has been computed for different values of the current, varying the latter from 10 to 40 A. The computations have been carried out using the approach in [19] for the construction with two air gaps and value of the second gap of 5 mm. 

**Figure 14:**
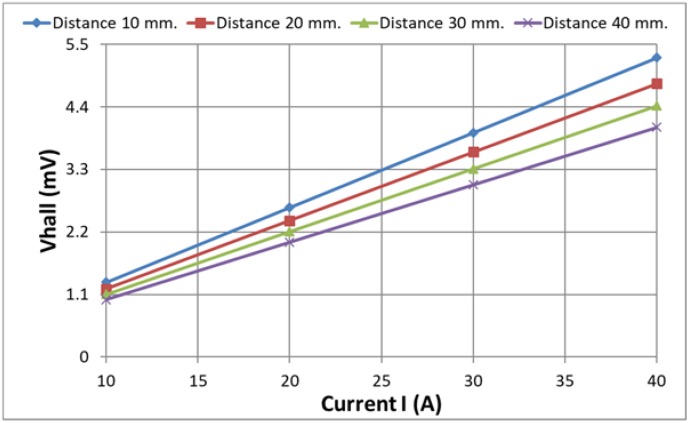
Dependence of the Hall plate voltage Vhall on the current for different distances to the conductor.

**Figure 15:**
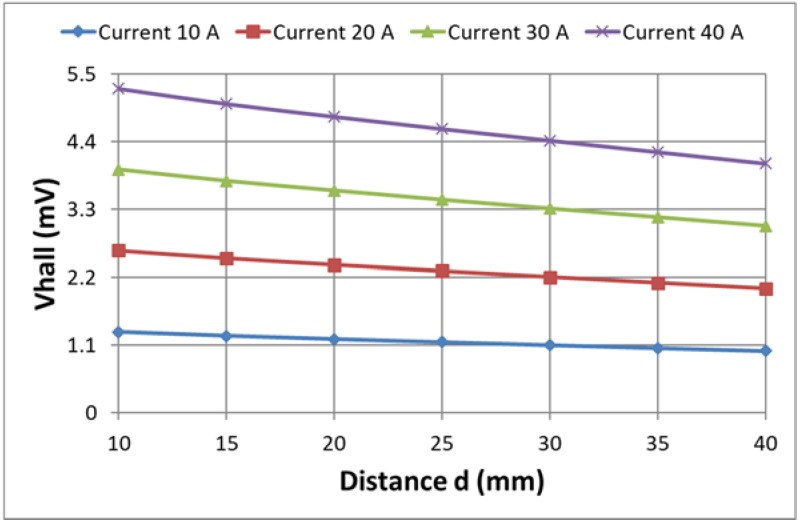
Dependence of the Hall plate voltage Vhall on the distance to the conductor for four different currents.

In Figure 14, the dependence of the Hall plate output voltage *Vhall* on the current *I* for different distances to the conductor is given. In Figure 15, the same family of curves has been presented as the dependence of the Hall plate output voltage on the distance to the conductor for different currents.

The obtained results give the possibility of estimating the maximal difference in the output signal when varying the distance from the conductor to the Hall plate from 10 to 40 mm. This maximal difference is less than 24% for all currents.

The linearity of the obtained dependencies could be used as an objective function in a future optimization. Having the best linear fit of the characteristics (Hall plate output voltage relative to the distance of the conductor to the Hall plate), it will be possible to design a current sensor with preliminary estimated current error with respect to the distance error.

## 5. Conclusions

Computer models for the constructions of Hall effect-based current sensors with additional open core magnetic concentrators are created. The employment of an open core magnetic concentrator leads to an almost linear dependence of the output signal on the distance between the conductor and the Hall plate. The influence of changing different geometric concentrator parameters is estimated for two constructions of the magnetic concentrator of rectangular shape, showing a more significant influence of the window length and height. The dependence of the Hall plate output voltage on the distance to the conductor has been obtained for different values of the current, and estimation of its linearity can be performed. This linearity can be the subject of further optimization with the aim of the design of a current sensor with an open core magnetic concentrator for nonfixed arrangements.
